# Tofacitinib: A Promising Therapeutic Option for Refractory Pityriasis Rubra Pilaris—A Case Report

**DOI:** 10.1002/ccr3.71232

**Published:** 2025-10-14

**Authors:** Mahsa Taremi, Nikoo Mozafari

**Affiliations:** ^1^ Faculty of Medicine Shahid Beheshti University of Medical Sciences Tehran Iran; ^2^ Skin Research Center Shahid Beheshti University of Medical Sciences Tehran Iran; ^3^ Department of Dermatology, Loghman Hakim Hospital Shahid Beheshti University of Medical Sciences Tehran Iran

**Keywords:** JAK inhibitors, pityriasis rubra pilaris, tofacitinib, upadacitinib

## Abstract

Pityriasis rubra pilaris (PRP) is a rare inflammatory skin disorder that can severely impact the quality of life in patients, particularly when presenting in chronic or refractory form. Emerging evidence suggests that JAK inhibitors may offer a potential treatment option. Here, we present a case of refractory PRP successfully treated with tofacitinib.

## 1 Introduction

Pityriasis rubra pilaris (PRP) is a rare, diverse inflammatory skin disorder of unknown cause, initially presenting as erythematous papules distributed in a follicular pattern. These papules subsequently coalesce to form plaques, often accompanied by the hallmark finding of “islands of sparing.” [1]. PRP can be categorized into six morphologic subtypes; Type I is the most common. The variant typically manifests with erythematous, scaly papules on the scalp and face, which may progress to involve the torso and extremities. Additional features include the characteristic waxy orange‐red palmoplantar keratoderma [1].

The chronic nature of this condition, coupled with its diagnostic and therapeutic challenges, significantly impacts patients' quality of life and elevates the risk of depression and anxiety [2].

Current therapeutic approaches for PRP are primarily based on clinical observations, case series, and individual case reports, as there is no established standard of care [3]. Treatment for PRP typically involves a multimodal approach, combining emollients, keratolytic agents, topical corticosteroids, and topical calcineurin inhibitors to manage cutaneous symptoms such as itching and burning. These topical therapies are often used in conjunction with systemic treatments to achieve optimal symptom control [3]. Systemic therapeutic options encompass retinoids and immunosuppressants such as methotrexate and cyclosporine [3]. In the management of refractory forms of PRP or in patients unresponsive or ineligible for conventional systemic therapies, multiple case studies have documented positive outcomes with the use of IL‐17A inhibitors, including ixekizumab and secukinumab, as well as IL‐23 inhibitors such as ustekinumab and guselkumab [4].

However, in instances where patients exhibit resistance to these treatments, the utilization of JAK inhibitors has been highlighted in a few reports as potential treatment options [5, 6, 7].

This case report highlights the management of a patient with PRP that was resistant to conventional therapies, including retinoids and methotrexate. Given the documented success of tofacitinib in a prior case report [7] and the unavailability of biologics such as IL‐17 or IL‐23 inhibitors in our country, we chose to administer tofacitinib to our patient. This approach yielded a favorable clinical response.

## 2 Case History/Examination

A 57‐year‐old female patient with no prior medical conditions visited the clinic due to an itchy skin lesion that had persisted for several months. Initially appearing on her face, the cutaneous eruptions progressed in a head‐to‐toe pattern, eventually affecting her extremities and the back of her neck. No other family members exhibited similar symptoms.

**FIGURE 1:**
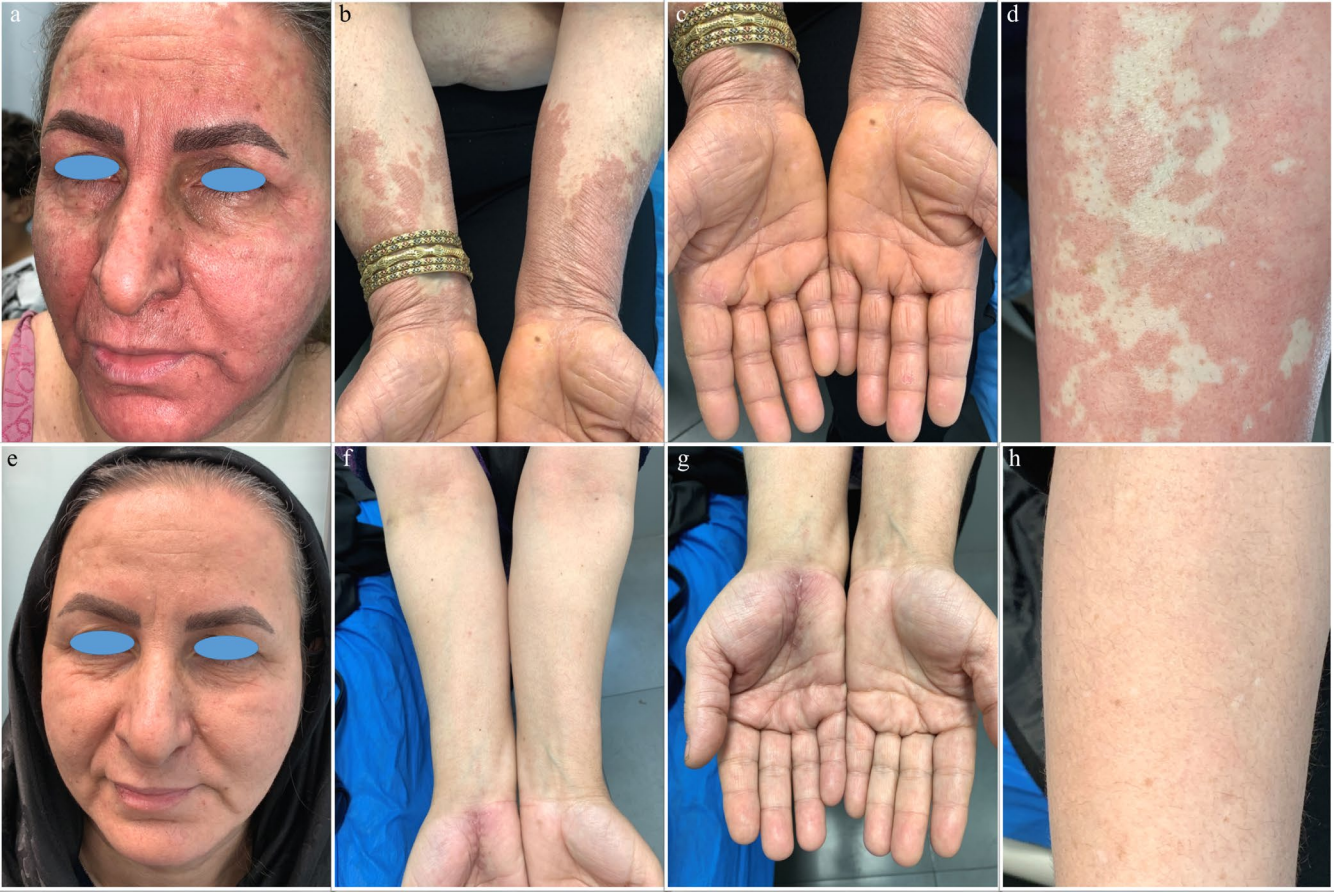
Before treatment: scaly, erythematous papules and plaques on the face and extremities, predominantly on the extensor surfaces (a–c). There were also some unaffected skin islands noted (d). After treatment with tofacitinib, a significant improvement in erythema and scaling was observed at the 6‐month follow‐up, (e–h).

Upon examination, various scaly, erythematous papules and plaques were observed on her face and extremities, predominantly on the extensor surfaces (Figure 1a–c). Some unaffected skin islands were also noted (Figure 1d). Scaly papules surrounding hair follicles were observed on the posterior neck region. Scalp examination revealed scaly plaques, and keratoderma was noted on the palms and soles. However, her mucous membranes, hair, and nails appeared normal. The clinical presentation pointed towards a potential diagnosis of either psoriasis or PRP.

## 3 Methods(Differential Diagnosis, Investigations and Treatment)

A 4 mm diameter punch biopsy of the affected skin was taken. The biopsy results indicated hyperkeratosis, psoriasiform acanthosis, alternating orthokeratosis and parakeratosis with follicular plugging, along with an infiltration of mixed lymphoplasmacytic cells. These microscopic findings confirmed the diagnosis of PRP.

Before initiating treatment, we conducted a comprehensive screening of the patient's liver and kidney function, hepatitis B, C, and HIV status, and PPD test to check latent tuberculosis, all of which yielded normal results with no significant abnormalities detected. Treatment was initiated with a daily 25 mg dose of Acitretin. After 2 months, due to an insufficient response to the initial treatment, 15 mg of Methotrexate with 5 mg folic acid was added to the regimen on a weekly basis. However, even after 2 months of therapy, the patient continued to experience persistent erythema, scaling, and itching. Taking into account the case reports [5, 7] highlighting the efficacy of tofacitinib in managing patients resistant to conventional treatments, Rhofanib (tofacitinib by Nanoalvand Corp, Iran) was introduced at a dosage of 5 mg twice daily, in addition to the existing therapy.

## 4 Conclusion and Results (Outcome and Follow‐Up)

Following 1 month of treatment, the patient reported a resolution of pruritus. A significant improvement in erythema and other signs was observed at the six‐month follow‐up (Figure 1e–h). Acitretin and methotrexate were gradually tapered and discontinued over 6 months, while tofacitinib was continued for an additional 6 months. The patient remained well controlled with no relapse. Subsequently, tofacitinib was tapered to 5 mg daily and has been maintained at this dosage to date.

## 5 Discussion

PRP can severely impact the quality of life in patients, particularly when presenting in chronic or refractory form [1]. Due to the infrequent occurrence of this condition and the lack of standardized studies, there is no established treatment protocol for PRP [3]. As a result, managing refractory forms remains a considerable challenge in clinical practice. The precise pathogenesis of PRP remains unclear; however, studies have demonstrated a significant increase in the expression levels of innate and adaptive cytokines, including TNF, IL‐12, IL‐23, IL‐17A, and IL‐22, within patent skin lesions [8].

The Janus kinase/signal transducers and activators of the transcription (JAK/STAT) signaling pathway play a critical role in modulating gene transcription for proinflammatory cytokines, thereby contributing to the pathogenesis of various immune‐mediated inflammatory disorders [9]. The advent of JAK inhibitors has sparked considerable interest in their potential application for managing various autoimmune disorders due to their capacity to inhibit a broad spectrum of cytokines [10]. This therapeutic approach has gained particular attention in the context of treating conditions such as psoriasis, alopecia areata, atopic dermatitis, and other related disorders [10]. In recent developments, the efficacy of JAK inhibitors in managing refractory PRP has been demonstrated [7].

Ying et al. reported a case study involving a 57‐year‐old female patient presenting with generalized scaly red plaques and papules. The patient was initially treated with a combination of acitretin (10–20 mg/d) and ixekizumab for approximately 3 months; however, the disorder continued to worsen. Subsequently, the patient was administered tofacitinib (JAK1 and JAK3 inhibitor) at a dosage of 5 mg twice daily. Following 4 weeks of treatment, a significant improvement was observed, and complete clearance of skin lesions was achieved after 3 months of follow‐up [7, 11].

Song et al. reported a case involving an 81‐year‐old female patient presenting with a chronic, widespread, erythematous, and scaly eruption, persistent for several years. The patient was initially administered acitretin (25 mg thrice weekly), urea 40% cream, and ixekizumab. However, after 12 weeks of treatment, no significant improvement was observed, leading to the discontinuation of all previous therapies. Subsequently, the patient was started on upadacitinib (selective JAK1 inhibitor) at a dosage of 15 mg daily. Following 4 weeks of treatment, a noticeable improvement in the patient's condition was reported [11].

Successful management of erythrodermic PRP with upadacitinib has also been documented. Li et al. reported two cases of erythrodermic PRP, both of which exhibited a partial response to treatment with secukinumab. Upon switching the therapy to upadacitinib (15 mg/day), a rapid and significant clearance of lesions was observed in both patients after 4 weeks of treatment [12].

The efficacy of tofacitinib in the treatment of PRP has been documented in the literature. Tan et al. reported a case involving a 39‐year‐old female patient who did not achieve significant improvement with conventional therapies, including topical corticosteroids and systemic acitretin. Biologic therapies such as secukinumab and ustekinumab were not pursued due to their high cost. Following the initiation of tofacitinib at a dose of 5 mg twice daily, the patient exhibited remarkable improvements in rash and pruritus within 1 week. This therapeutic response persisted over 2 months of treatment without any reported adverse effects. Notably, a 2‐month follow‐up post‐discontinuation of tofacitinib showed no recurrence of symptoms, highlighting its potential as an effective treatment for refractory PRP [5].

Similar to reported cases, in this case report, we present a patient who was initially administered systemic retinoid therapy, which proved to be ineffective. Subsequently, methotrexate was added to the treatment regimen, yet the response remained inadequate. A significant improvement in the patient's condition was observed upon initiating tofacitinib. No adverse effects were reported throughout the entire course of therapy.

In conclusion, this case report supports the potential of tofacitinib as a treatment option for patients with refractory PRP.

## Author Contributions

**Mahsa Taremi:** writing – original draft. **Nikoo Mozafari:** conceptualization, data curation, writing – review and editing.

## Disclosure

We declare that none of the authors listed on the manuscript are employed by a government agency that has a primary function other than research and/or education, and none of the authors submitting this manuscript are as an official representative or on behalf of the government.

## Consent

The patient in this manuscript gave written informed consent for the publication of her case details.

## Conflicts of Interest

The authors declare no conflicts of interest.

## Data Availability

The data presented in this study is available on request from the corresponding author.
